# Changing Paradigms in the Diagnosis of Ischemic Heart Disease by Multimodality Imaging

**DOI:** 10.3390/jcm11030477

**Published:** 2022-01-18

**Authors:** Andrea Baggiano, Gianpiero Italiano, Marco Guglielmo, Laura Fusini, Andrea Igoren Guaricci, Riccardo Maragna, Carlo Maria Giacari, Saima Mushtaq, Edoardo Conte, Andrea Daniele Annoni, Alberto Formenti, Maria Elisabetta Mancini, Daniele Andreini, Mark Rabbat, Mauro Pepi, Gianluca Pontone

**Affiliations:** 1 Cardiovascular Imaging Department, Centro Cardiologico Monzino IRCCS, 20138 Milan, Italy; andrea.baggiano@cardiologicomonzino.it (A.B.); gianpieroitaliano@gmail.com (G.I.); marco.guglielmo@cardiologicomonzino.it (M.G.); laura.fusini@cardiologicomonzino.it (L.F.); maragna.riccardo@gmail.com (R.M.); carlo.giacari@gmail.com (C.M.G.); saima.mushtaq@cardiologicomonzino.it (S.M.); edoardo.conte@cardiologicomonzino.it (E.C.); andrea.annoni@cardiologicomonzino.it (A.D.A.); alberto.formenti@cardiologicomonzino.it (A.F.); maria.mancini@cardiologicomonzino.it (M.E.M.); daniele.andreini@cardiologicomonzino.it (D.A.); mauro.pepi@cardiologicomonzino.it (M.P.); 2 Cardiovascular Section, Department of Clinical Sciences and Community Health, University of Milan, 20122 Milan, Italy; 3 Department of Emergency and Organ Transplantation, Institute of Cardiovascular Disease, University Hospital Policlinico of Bari, 70124 Bari, Italy; andrea.guaricci@gmail.com; 4 Department of Biomedical Sciences for Health, University of Milan, 20133 Milan, Italy; 5 Division of Cardiology, Department of Medicine and Radiology, Loyola University of Chicago, Chicago, IL 60660, USA; mrabbat@lumc.edu; 6 Division of Cardiology, Department of Medicine, Edward Hines Jr. VA Hospital, Hines, IL 60141, USA

**Keywords:** coronary artery disease, echocardiography, single-photon emission computed tomography, positron emission tomography, magnetic resonance, computed tomography angiography, fractional flow reserve, myocardial perfusion, clinical management

## Abstract

Coronary artery disease (CAD) represents the most common cardiovascular disease, with high morbidity and mortality. Historically patients with chest pain of suspected coronary origin have been assessed with functional tests, capable to detect haemodynamic consequences of coronary obstructions through depiction of electrocardiographic changes, myocardial perfusion defects or regional wall motion abnormalities under stress condition. Stress echocardiography (SE), single-photon emission computed tomography (SPECT), positron emission tomography (PET) and cardiovascular magnetic resonance (CMR) represent the functional techniques currently available, and technical developments contributed to increased diagnostic performance of these techniques. More recently, cardiac computed tomography angiography (cCTA) has been developed as a non-invasive anatomical test for a direct visualisation of coronary vessels and detailed description of atherosclerotic burden. Cardiovascular imaging techniques have dramatically enhanced our knowledge regarding physiological aspects and myocardial implications of CAD. Recently, after the publication of important trials, international guidelines recognised these changes, updating indications and level of recommendations. This review aims to summarise current standards with main novelties and specific limitations, and a diagnostic algorithm for up-to-date clinical management is also proposed.

## 1. Introduction

Coronary atherosclerosis is a dynamic multifocal process characterized by plaque accumulation and subsequent functional changes of coronary circulation. Atherosclerotic disease starts as intimal thickening spontaneously after birth, providing a soil for initial lesion growth [1] and the progression to macroscopic entities usually takes several decades, thus possibly being clinically relevant in subjects over 35 years of age [2]. Coronary artery disease (CAD) development can be negatively influenced by commonly known risk factors, such as uncontrolled hypertension, dyslipidaemia, diabetes, visceral obesity, smoking habit, genetic predisposition, while a favourable influence is related to regular physical activity, ideal body weight and a balanced Mediterranean diet. Once disease has led to macroscopic changes, effective interventions can be performed either non-invasively, through a pharmacological approach [3], or invasively, through revascularization.

The common scenario in which the presence of CAD is investigated is in the setting of newly developed chest pain. Once the presence of acute coronary syndrome is ruled out, usually during emergency department (ED) evaluation [4], physicians are typically requested to assess such patients on an outpatient basis. Several scientific guidelines/position papers related to this topic have been delivered by national and international societies, and regularly updated over the last decade. Without any doubt, along with the 2016 update of National Institute for Health and Care Excellence (NICE) guidelines for the management of chest pain of recent onset [5], the 2019 European Society of Cardiology (ESC) Guidelines on Chronic Coronary Syndromes [6] have substantially contributed to a change of practice. Indeed, the latest European guidelines updated pre-test probability estimation of obstructive CAD according to evidence from landmark trials such as CONFIRM, PROMISE and the retrospective observational analysis from Copenhagen [7,8,9], and upgraded coronary computed tomography angiography (cCTA) to class I indication as the initial test to diagnose CAD, equalling the strength given to stress imaging. Finally, these guidelines downgraded exercise ECG to level IIb, now recommended for the diagnosis of obstructive CAD in naïve patients only when there is no adequate expertise/access to imaging techniques [6].

Physicians involved in the investigation of new onset chest pain act with the goal of preventing future major acute cardiac events (MACE) such as death or myocardial infarction, and the first step is estimating the pre-test likelihood of obstructive CAD according to age, gender, chest pain characteristics and prevalence of the disease in the studied population. Usually, this stage does not require further investigations when the pre-test probability is very low (i.e. <5%), in order to avoid unnecessary diagnostic tests. In cases of higher likelihood, testing may be considered (between 5 and 15%, especially with the coexistence of modifiers such as the presence of several risk factors for cardiovascular disease, resting ECG changes or myocardial dysfunction), or is considered most beneficial (>15%) [6]. Thus, the choice of non-invasive test prescription is crucial in clinical management.

The scientific evidence behind such recommendation updates and continuous technical development has led to changes in approaching the clinical setting of suspected ischaemic heart disease, and this review will summarise current standards with main novelties and specific limitations, and a diagnostic algorithm for an up-to-date clinical management will be proposed.

## 2. Last Updates Regarding Available Second-Line Non-Invasive Tests

The novelty introduced by the 2019 ESC Guidelines on Chronic Coronary Syndromes in terms of CAD detection was to assign the same level of recommendation to non-invasive diagnostic techniques. On one hand, cCTA has the aim of displaying the presence and entity of coronary atherosclerosis; on the other, stress techniques are intended to show the consequence of obstructive atherosclerotic burden detecting the presence of inducible ischaemia.

Historically, non-invasive assessment of suspected CAD has relied on functional techniques. One of the most prescribed functional tests is exercise electrocardiography (Ex-ECG) due to its wide availability and simple reporting [10]; however, overall diagnostic accuracy has been proven to be limited for the detection of obstructive CAD [11], especially in specific settings such as female gender [12] and ECG resting abnormalities [13]. In light of the poor overall diagnostic performance, this approach has formally been downgraded to a lower level of recommendation [6].

According to availability, centre expertise, and patient characteristics, recommended functional non-invasive tests for the diagnosis of obstructive CAD are the techniques able to detect myocardial ischaemia through stress perfusion abnormalities or stress wall motion abnormalities. Thanks to the evaluation beyond ECG changes, tests such as stress echocardiography, SPECT, PET and stress CMR are associated with better accuracy for the detection of flow-limiting coronary stenosis compared with invasive functional testing through fractional flow reserve (FFR) [14].

### 2.1. Stress Echocardiography

Stress echocardiography (SE) is one of the second-line stress tests used for the assessment of patients presenting with chest pain and intermediate pre-test likelihood of obstructive CAD [15]. This technique is characterised by low cost, absence of ionizing radiation, ease of performance [16], good diagnostic results [17] and excellent prognostic implications, either performed with physical exercise or a pharmacological (inotropic or vasodilator) stressor [18,19,20]. However, especially when only regional wall motion abnormalities (RWMA) are investigated, suboptimal images due to poor acoustic windows and submaximal stress are important factors affecting diagnostic accuracy [21]. In order to overcome such limitations, new approaches with the addition of parameters with different pathophysiological targets have been proposed [22].

**Figure 1:**
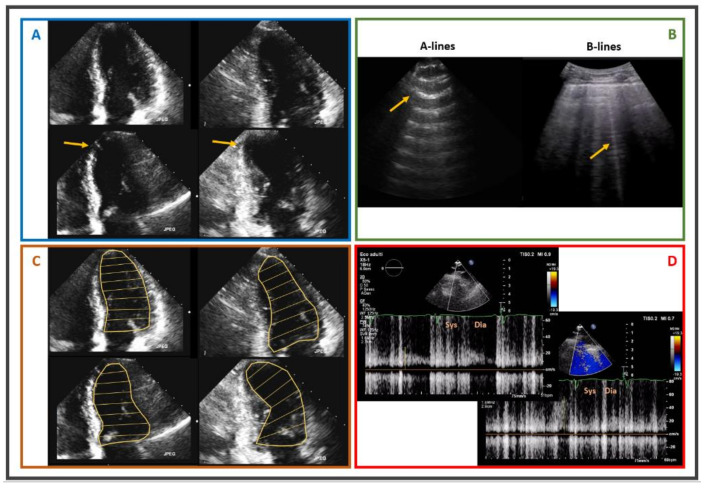
Exercise stress echocardiography performed in a 58-year-old male, smoker, with hypertension, who recently developed exertional chest pain. Panel (**A**): upper images show 4Ch and 2Ch views with normal kinesis at rest, while lower panels show 4Ch and 2Ch views under stress, characterised by apical septal akinesis. Panel (**B**): B-lines in lung ultrasound during stress. Panel (**C**): upper images show 4Ch and 2Ch views with normal LV size at rest, while lower panels show 4Ch and 2Ch views under stress, characterised by dilated end-systolic volume. Panel (**D**): blunted increase at peak stress of pulsed-wave Doppler diastolic flow at mid LAD. Patient was referred to ICA, pathological for critical stenosis of the mid LAD. *4Ch: four chambers; 2Ch: two chambers; LV: left ventricle; LAD: left anterior descending; ICA: invasive coronary angiography; Sys: systolic; Dia: diastolic.*

An attempt to strengthen the diagnostic and prognostic role of SE is the development of the ‘ABCD’ protocol. This approach is characterised by the simultaneous evaluation of B-lines during stress phase (early event of pulmonary interstitial oedema) [23], left ventricular contractile reserve and global myocardial function (expression of the intrinsic contractile state of left ventricle) [24,25] and coronary flow velocity reserve of the left anterior descending coronary artery [26]. A clinical case with a complete ‘ABCD’ protocol is displayed in Figure 1.

Further optimisation of this protocol has been proposed, with the upgrade to ‘ABCDE’ [27], where letter E accounts for imaging-independent ECG-based heart rate reserve (HRR) [28]. With this approach, Ciampi et al recently demonstrated in a prospective, multicentre, international, effectiveness study of 3574 patients that a score derived from this comprehensive assessment is able to precisely stratify prognosis [29].

### 2.2. Single-Photon Emission Computed Tomography

Stress myocardial perfusion imaging (MPI) with SPECT represents one of the most currently prescribed tests for ischemia detection [30]. The diagnostic role of this imaging modality is certainly established, and a negative SPECT identifies subjects at low risk of MACE [31]. However, traditional SPECT systems use large sodium iodide crystals, photomultiplier tubes, and parallel-hole collimation, with relevant drawbacks such as prolonged imaging times and relatively large radioisotope doses. Furthermore, overall diagnostic accuracy, having invasive functional assessment as reference standard, has been shown to be lower than desirable [14,21,32].

Recently, SPECT-MPI has undergone major technological development in order to increase diagnostic and prognostic performance. Mostly, advances were possible through implementation of cadmium zinc telluride (CZT) solid-state detectors, specialized collimators, and software-based resolution recovery [33,34], noticeably increasing sensitivity and image quality [35].

In the WATERDAY Study, Agostini et al assessed the feasibility of myocardial blood flow (MBF) and flow reserve (MFR) estimation using dynamic CZT SPECT in patients with stable CAD, in comparison with ^15^O-water PET and fractional flow reserve (FFR). In thirty patients, authors found that CZT SPECT yielded similar MFR in global and each vessel territory compared to PET, having high diagnostic accuracy for the detection of ischaemia and hemodynamically significant stenoses [36]. Another experience from Giubbini et al. showed that myocardial blood flow reserve (MFR) estimation with ^99m^Tc-tetrofosmin-CZT SPECT, optimised with the addition of an attenuation correction, demonstrated a good linear regression compared to quantitative assessment with PET, and a good ability to predict pathological MFR and stress MBF [37].

However, a review recently published from Renaud et al showed that imprecision vs PET MFR ranged from 0.556 to 0.829, and test-retest imprecision was 0.781–0.878. Simulations used to evaluate the impact of SPECT MFR imprecision on confidence of clinically relevant categorization showed correct classification of up to only 34% of patients when true MFR value was between 1.5 and 2.0, with high confidence (>80%) only achieved for extreme MFR values (<1.0 or >2.5), and correct classification in only 15% of patients in a typical scenario with MFR of 1.8 ± 0.5 [38].

Finally, another experience from Zavadovsky et al, analysing the state-of-the-art MBF assessment with CZT SPECT and technical issues related to image acquisition, data post-processing and interpretation, concluded that standardization of acquisition and post-processing protocols are needed in order to reduce inter-sites variability and increase the clinical relevance of CZT SPECT results [39].

### 2.3. Positron Emission Tomography

Absolute quantification of myocardial blood flow (MBF) by positron emission tomography (PET) is a very good technique in obviating the underestimation of myocardial ischaemia, especially in challenging settings such as with multi-vessel or left-main disease [40,41]. Several recent meta-analyses on the diagnostic performance of non-invasive tests showed how quantitative myocardial perfusion assessment by PET is the most effective approach in detecting functionally relevant CAD [14,21]. Several tracers can be used in this clinical context, with ^82^Rubidium (^82^Rb) and ^13^N-Ammonia being the most widely used. However, due to its intrinsic nature, PET with ^15^O-water tracer, characterised by metabolic inertia, passive diffusion into the myocardium, excellent first-pass extraction independent of flow rate, and regional uptake directly proportional to segmental MBF, is considered the gold standard technique of non-invasive MBF measurement [40,42].

Moreover, when CAD and left ventricle systolic dysfunction coexist, the assessment of myocardial glucose metabolism with ^18^F-fluorodeoxyglucose (^18^F-FDG) PET allows sensitive detection of viable tissue, with potential for improvement of systolic function after revascularization [43,44,45].

Unfortunately, relevant practical factors such as limited expertise and availability of scanners and tracers, and related high costs represent an important limitation to a wide and routine use of this high-performance technique [46].

### 2.4. Cardiovascular Magnetic Resonance

Cardiovascular magnetic resonance (CMR), thanks to excellent spatial, temporal and contrast resolution, represents one of the most effective non-invasive functional tests for the assessment of functionally relevant CAD [47,48,49,50], with a solid role from both diagnostic and prognostic perspectives [51,52,53]. In the MR-INFORM (Magnetic Resonance Perfusion or Fractional Flow Reserve in Coronary Disease) trial, Nagel et al. showed that a non-invasive imaging approach with stress perfusion CMR was not inferior to invasive assessment with fractional flow reserve (FFR) in planning appropriate invasive procedures with respect to future cardiac events [48]. Stress CMR allows for the detection of perfusion defects after short half-life vasodilator stress agent administration, such as adenosine; perfusion defects and regional wall motion abnormalities after long half-life vasodilator stress agent administration, such as dipyridamole and regadenoson; or regional wall motion abnormalities after inotropic agent administration such as dobutamine [49]. Preliminary experiences are also focusing on ischemia assessment during physical exercise using an MRI compatible ergometer bicycle, with promising results [54,55].

**Figure 2:**
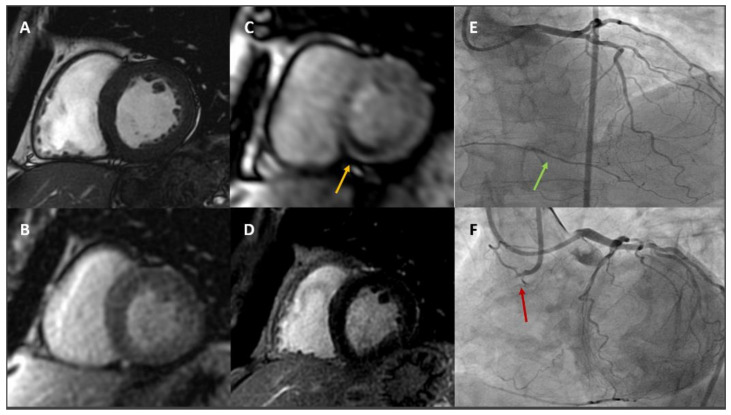
A 62-year-old male with hypertension and dyslipidaemia developed exertional dyspnoea and stress CMR was prescribed. Panel (**A**): basal LV SAx cine sequence showing basal LV segments with normal wall thickness and kinesis. Panel (**B**): basal LV SAx perfusion sequence showing normal basal myocardial perfusion at rest. Panel (**C**): basal LV SAx perfusion sequence showing severe hypoperfusion at basal inferoseptum and inferior wall after adenosine administration (yellow arrow). Panel (**D**): basal LV SAx LGE sequence showing normal myocardial viability. Panel (**E**): ICA showing absence of severe stenoses at LAD and LCx, while collaterals from distal LAD to RCA are noted (green arrow). Panel (**F**): ICA showing ostial RCA CTO (red arrow). *CMR: cardiovascular magnetic resonance; LV: left ventricle; SAx: short axis; LGE: late gadolinium enhancement; ICA: invasive coronary angiography; LAD: left anterior descending; LCx: left circumflex; RCA: right coronary artery; CTO: chronic total occlusion.*

Furthermore, this technique allows, after gadolinium-based contrast administration, detailed assessment of myocardial viability and scar detection with late gadolinium enhancement (LGE) imaging, thus obtaining further prognostic information [56], and helping the physicians in performing the patient’s ideal clinical and invasive management [57]. Figure 2 shows a clinical case in which viability and ischaemia assessment guided clinical management.

To further increase the diagnostic accuracy of perfusion defect evaluation, usually performed on visual assessment, recently quantitative approaches through pixelwise quantitative perfusion mapping has been developed. Interestingly, this tool increased diagnostic accuracy in perfusion defect detection, especially in the setting of multivessel disease or microvascular dysfunction [58,59].

However, despite a favourable clinical profile, stress CMR represents one of the most expensive non-invasive tests, suffers from long scanning time and clinical limitations (claustrophobia, metal devices, patients with clinical instability and life-threatening arrhythmias, etc.).

### 2.5. Cardiac Computed Tomography Angiography and Plaque Imaging

Moving from functional to anatomical assessment, cCTA has been validated as the only non-invasive imaging test able to describe coronary atherosclerosis, pathological key element leading to myocardial infarction and stable angina, even in a non-obstructive phase, with both a good safety profile and high diagnostic accuracy [60,61,62,63].

While CT calcium scoring has provided a surrogate of coronary plaque burden for many years [64], nowadays a precise quantification of both calcified and non-calcified components of coronary atherosclerosis is possible thanks to dedicated semi-automated and automated tools [3].

**Figure 3:**
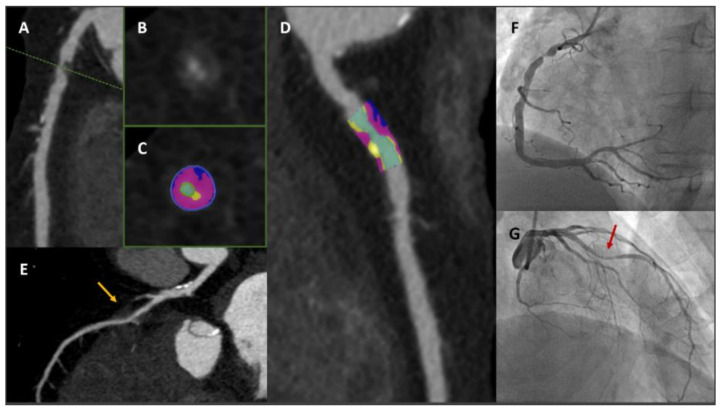
A 75-year-old male with diabetes and dyslipidaemia developed typical chest pain and cCTA was prescribed. Panels (**A**–**D**): critical stenosis with high-risk plaque features at proximal RCA. Panels (**B**,**C**): cross-section images at most stenotic level (green dotted line). Panels (**C**) (short axis) and (**D**) (long axis) with advanced plaque analysis: residual lumen identified with colour green, fibrofatty component (<30 HU) identified with colour blue, fibrotic component (between 30 and 350 HU) identified with colour purple, calcific component (>350 HU) identified with colour yellow. Panel (**E**): LAD characterised by non-significant calcific disease at proximal segment and deep myocardial bridge at mid segment (yellow arrow). Panels (**F**,**G**): ICA showing critical stenosis at proximal RCA (Panel (**F**)) and deep myocardial bridge at mid LAD (Panel (**G**), red arrow). *cCTA: cardiac computed tomography angiography; RCA: right coronary artery; HU: Hounsfield unit; LAD: left anterior descending; ICA: invasive coronary angiography.*

cCTA is able to qualitatively and quantitatively assess also specific features defined as ‘adverse plaque’ phenotype, including low-attenuation plaque (a marker of a large necrotic core), positive remodelling (outer vessel diameter greater than 10% of the diameter of the reference normal segment within the same vessel), spotty calcification (focal calcifications with diameter less than 3 mm), and the ‘napkin ring’ sign (inner area of low attenuation surrounded by a rim of higher attenuation) [65,66,67,68,69]. Figure 3 shows a clinical case in which cCTA provides a precise description of both stenosis entity and plaque features.

Detailed plaque features are not pure radiological findings, rather, extensive data regarding the strong relationship between plaque characteristics and MACE have been collected. The ICONIC Study showed that nearly three-quarter of acute coronary syndromes are related to events involving non-obstructive stenoses [70]. Similar evidence is provided by PROMISE Trial, in which the presence of adverse plaque phenotype was associated with 70% increase of major events such as death, myocardial infarction and hospitalization for unstable angina [71,72]. Furthermore, SCOT-HEART Study highlighted the prognostic importance of plaque burden characterisation beyond common tools such as cardiovascular risk scores, coronary artery calcium score, or coronary artery stenosis severity. While death or nonfatal myocardial infarction was 3 times more frequent in patients with plaques with adverse features and twice as frequent in those with obstructive disease, patients with both obstructive disease and adverse plaque features had the highest event rate, with a 10-fold increase in MACE compared with patients with normal coronary arteries [73]. Moreover, while low-attenuation plaque burden correlated weakly with cardiovascular risk scores but strongly with the severity of luminal coronary stenosis, low-attenuation plaque resulted the strongest predictor of myocardial infarction, irrespective of cardiovascular risk score, CAC score, or lumen area stenosis [74]. Notably, another interesting evidence is provided by EMERALD study, in which Lee et al showed that analysis of adverse hemodynamic plaque characteristics (defined as forces acting on plaque walls, such as change of fractional flow reserve derived by cCTA across lesion, wall shear stress, and axial plaque stress) combined with description of ‘high-risk’ plaque features improved the identification of culprit lesions related to acute coronary events [75].

Finally, cCTA can be used to describe change in plaque volume and composition as a consequence of medications. Lee et al showed in PARADIGM study that patients regularly treated with statin where characterized at serial cCTA scan by slower progression over time of non-calcified plaques and increased conversion of non-calcified to calcified plaque [3]. Recently, van Rosendael et al used on 2458 coronary lesions from 857 patients a refined plaque categorization based on six different range of attenuation, and showed that untreated coronary lesions increased in volume over time for all 6 compositional types, while statin therapy was associated with volume decreases in low-attenuation plaque, fibro-fatty plaque and greater progression of high-density calcium plaque [76]. The transformation of non-calcified to high-density calcium plaque could be seen as one of the most favourable effect of statin at coronary level. In a multicentre case-control cohort study with individuals from ICONIC study and CONFIRM registry, presence of high-density calcium plaque was associated with a lower risk for future acute coronary events [77].

However, some drawbacks of cCTA need to be acknowledged. Mostly, unavoidable radiation exposure and the risk of overestimation of coronary stenosis, with the potential referral to unnecessary invasive angiography, limit widespread use of this technique. Moreover, detailed plaque burden quantification and characterisation, though use of automated and semi-automated software, is still time-consuming, and hardly implemented in daily routine. However, thanks to technical developments, radiation exposure has been dramatically reduced, from 5–6 mSv to an average of 1–2 mSv [78], and overestimation of vessel stenoses, mainly due to calcific burden, has been reduced [79].

## 3. Function vs. Anatomy

Ideally, all non-invasive tests could be pooled into two different groups, characterised by two different aims: on one hand, functional non-invasive tests look for ischaemia detection; on the other, cCTA identifies coronary atherosclerosis. The last decade of research in this field contributed significantly to outlining the preferable approach.

The prospective multicentre EVINCI study enrolled 697 patients with chronic chest pain and intermediate probability of having CAD according to the updated Diamond and Forrester score from 17 European centres. In this trial, largely based on the application of currently available non-invasive tests, patients with positive tests underwent heart catheterization (coronary angiography and invasive functional measurements when appropriate) as a reference method to define the presence and extent of functionally significant CAD. The novel result of the EVINCI study was the clear demonstration of superiority of cCTA for diagnosing the presence of obstructive CAD in this specific population of patients with low-intermediate prevalence of disease [80]. As secondary end-points, the outcome analysis of the EVINCI data showed that a combined anatomical-functional non-invasive screening of patients avoided unnecessary invasive procedures, only referring patients to ICA with the combination of positive cCTA and stress test, translating into a positive impact on MACE reduction and cost-effectiveness [81,82].

The multicentre, prospective, open-label SCOT-HEART trial randomised a total of 4146 patients referred for the assessment of suspected angina from several Scottish centres to clinical care alone or clinical care plus addition of cCTA between 2010 and 2014, with the primary endpoint of the certainty of the diagnosis of angina due to CAD. At short-term follow-up, cCTA significantly reclassified the diagnosis of CAD and of angina due to CAD. The increased certainty in the diagnosis led to significant changes in planned investigations and treatments [83]. The favourable impact of early recognition of CAD, either non-obstructive or obstructive, coupled with the correct prescription of medical therapy (i.e. antiplatelet drugs and statins) and appropriate revascularization, led to a significantly lower rate of death from CAD or nonfatal myocardial infarction at 5-year follow-up compared to standard care alone [84]. A post-hoc analysis of the SCOT-HEART long follow-up study showed that beyond the first year, rates of coronary revascularization were higher in those who had received standard care alone, many of which triggered by myocardial infarction, suggesting that standard care may be associated with missed diagnoses of coronary atherosclerosis [85]. Apart from appropriate referral to invasive assessment, a key role was played by pharmacological therapies that decreased in the standard care group after initial assessment while markedly raised in the cCTA group. Indeed, in patients with comparable 10-year cardiovascular risk scores, the rates of antiplatelet and statin therapy use were nearly 3-fold higher in those with CAD compared with those without disease, highlighting that cCTA is extremely crucial for a “precision medicine” approach [85]. 

Such evidence reinforced the concept that the early detection of coronary atherosclerosis through cCTA and the non-invasive detailed analysis of plaque burden significantly affect both diagnosis and patient’s prognosis.

The ISCHEMIA trial was a multicentre randomised trial with the intent to establish whether early surgical or percutaneous revascularization was superior to medical therapy alone in preventing mortality and other MACE in patients with proven relevant coronary atherosclerosis and moderate-to-severe inducible ischaemia. In 5179 patients with optimized medical therapy, the invasive strategy was not superior to the conservative approach in terms of MACE (CV death, non-fatal myocardial infarction, unstable angina, heart failure) [86]. Interestingly, while there was no difference in cardiovascular and total mortality at 4-year follow-up, patients treated invasively had better results in controlling angina, took fewer antianginal drugs with increased utilization of DAPT. However, while patients undergoing the conservative strategy had greater “spontaneous” coronary events (Type I and II), patients treated invasively had a greater number of procedural-related coronary events [86].

The ISCHEMIA Trial also sought to determine whether estimates of severity of CAD and/or ischemia were independently associated with increased risk of all-cause mortality, myocardial infarction or other cardiovascular outcomes. According to the analysis of the 2475 subjects with established coronary atherosclerosis, CAD severity (defined as atherosclerosis extent and stenosis severity based on the modified Duke prognostic index) was a highly significant predictor of all endpoints (all-cause mortality, MI, CV death, hospitalization for unstable angina, heart failure or resuscitated cardiac arrest), with a good performance in stratifying prognosis according to graded disease severity. This classification was independent of ischaemia severity while, in contrast, ischaemia severity was associated with only MI [87]. These results are in line with the ones from SCOT-HEART, with anatomic assessment superior to predict events compared to ischemia detection.

In addition, it is worth considering another aspect strengthening the anatomical approach. The assessment of CAD severity in the ISCHEMIA Trial, though superior to ischaemia detection, was based on outdated use of stenosis severity, probably a relevant choice considering a trial based on ischemia and coronary revascularization, but surely less strong in terms of independent prediction of clinical events if compared to the modern approach of detailed plaque burden characterisation. Studies have demonstrated that most MI and related CV deaths are caused by nonobstructive plaques with high-risk plaque features [88,89,90]; that’s why a greater differential effect would have probably been obtained if these measures of atherosclerotic disease were used.

## 4. Function and Anatomy in a “One-Stop-Shop” Exam: CT-Derived Fractional Flow Reserve and CT Perfusion

In light of the recent above-mentioned scientific evidence, anatomical assessment with cCTA seems to be preferred in the evaluation of patients with suspected CAD. However, especially in patients with coronary atherosclerosis of intermediate severity and persistent symptoms despite optimal medical management, demonstration of functional relevance of coronary stenoses can be helpful in guiding revascularization.

It’s now possible to obtain information regarding functional relevance of coronary stenoses on top of detailed atherosclerotic burden description with CT. Two different methods have been developed and validated over the last decade, CT-derived Fractional Flow Reserve (FFR_CT_) and CT perfusion.

The FFR_CT_ technique has been developed to reproduce the acquisition of functional information similar to those obtained through ICA and FFR wire after adenosine administration. This tool permits the non-invasive calculation of FFR values for all major epicardial coronary arteries through a three-dimensional representation of the coronary tree derived from the cCTA dataset and then the application of computational flow dynamic algorithms under simulated hyperaemic flow [91,92]. Although on-site analysis systems have been developed, currently HeartFlow (HeartFlow Inc., Redwood, CA, USA) is the only commercial software approved for clinical use. Main advantages of this tool are that no additional scan data or pharmacological stressors are required, with the final report available at the referring site in less than 3 hours [93]. After initial validation against invasive FFR [94], and software optimisation in order to reduce false positives in highly calcific disease [95], clinical implication of FFR_CT_ has been assessed in multicentre studies. The PLATFORM (Prospective LongitudinAl Trial of FFR_CT_) study randomised 584 patients suspected of stable CAD to either a cCTA + FFR_CT_-driven strategy or to the usual care strategy. This trial showed that a significant reduction of approximately 61% of ICA was obtained when FFR_CT_ was added to cCTA, without negative effect in terms of clinical outcomes at 1-year follow-up [96]. Prospective, observational, and real-world clinic trials confirmed results from PLATFORM and demonstrated the safe deferral of unnecessary invasive coronary angiography in patients with stable CAD, with a high proportion of those who underwent invasive coronary angiography undergoing revascularization [97]. The ADVANCE registry enrolled 5083 patients referred for a clinically indicated cCTA for suspected CAD, and all patients enrolled received FFR_CT_ analysis. After incorporating information from FFR_CT,_ the clinical management was modified in two out of three patients, leading to an important reduction in invasive diagnostic tests, revascularizations, and adverse clinical events (heart attacks and deaths) at 90 days [98]. At mid-2021 the PRECISE (Prospective Randomized Trial of the Optimal Evaluation of Cardiac Symptoms and Revascularization) trial completed enrollment of 2100 patients, and results regarding the possible role of the combination of the PROMISE Risk Tool with cCTA and selective FFR_CT_ in improving outcomes over usual care are awaited.

Stress CT perfusion (CTP), unlike FFR_CT_, unmask the presence of inducible ischaemia secondary to the administration of a hyperaemic stimulus. Altering the physiological constant pressure gradient of the coronary circulation available in resting conditions, the presence of obstructive coronary stenosis induces a progressive reduction in coronary flow and myocardial perfusion; the latter depicted as reduced density zones [99,100]. The evaluation of stress myocardial perfusion is performed after adenosine administration, which can eventually either precede or follow cCTA (i.e. the rest phase) according to patient’s risk profile and physician’s preference [101]. Stress CTP images can be acquired through static or dynamic protocols, with the former characterised by a single stress dataset acquisition and pure qualitative assessment, and the latter characterised by the acquisition of multiple datasets following the kinetics of contrast in the cardiac chambers and a calculation of the myocardial blood flow (MBF) for each myocardial segment [102,103]. The most appealing aspect of dynamic stress CTP is its quantitative approach, which makes reporting less operator-dependent and more reproducible compared to static stress CTP, especially in challenging settings such as multivessel obstructive coronary disease or microcirculation dysfunction. However, compared to static CTP, the radiation exposure of a dynamic approach is higher, ranging between 8–9 mSv for “shuttle-mode” technique and 5 mSv for “whole-heart coverage” scanners. From a diagnostic point of view, experience from the PERFECTION (Stress Computed Tomography Perfusion Versus Fractional Flow Reserve CT Derived in Suspected Coronary Artery Disease) study revealed that both static and dynamic CTP increased diagnostic accuracy on top of cCTA in detecting functionally relevant coronary stenoses [104,105]. In terms of patient management, Lubbers et al showed that a CT strategy with possible use of CTP was competitive in terms of downstream non-invasive testing, accurate detection of stenoses subsequently treated with revascularisation and short-term outcome compared to a functional approach [106].

A direct comparison between FFR_CT_ (provided by HeartFlow) and Stress CTP in terms of diagnostic accuracy was conducted in the PERFECTION Study. Pontone et al showed a comparable accuracy between integrated protocols of cCTA + FFR_CT_ and cCTA + static CTP, at both per vessel and per patient levels. However, specificity and positive predictive values were slightly in favour of stress CTP, probably due to its more physiological nature, suggesting the clinical utility of a sequential strategy [105,107].

**Figure 4:**
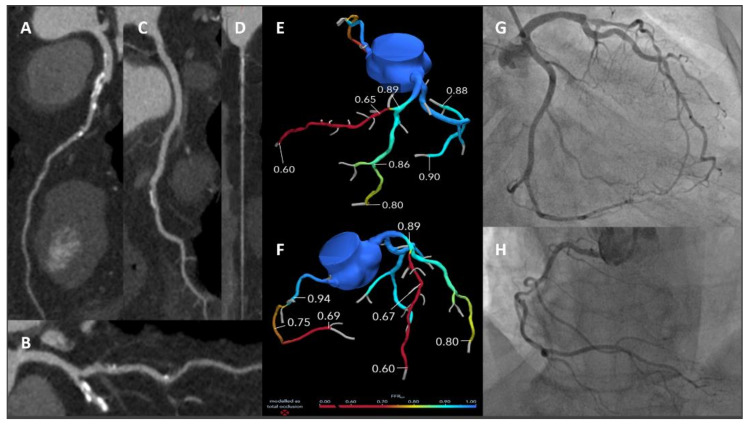
A 59-year-old male smoker with hypertension and dyslipidaemia presented with several episodes of atypical chest pain. A cCTA was prescribed. Panels (**A**–**D**) showed critical stenosis at the mid LAD (**A**), moderate stenosis at the main diagonal branch (**B**), mild calcific stenosis at the main obtuse marginal branch (**C**) and significant stenosis at the mid RCA (**D**). Panels (**E**,**F**): FFR_CT_ analysis (HeartFlow Inc.) showing haemodynamically significant LAD and RCA stenoses. Panels (**G**,**H**): ICA showing severe stenosis at the mid LAD, mild stenosis of the main diagonal branch and main obtuse marginal branch (**G**) and significant stenosis of the mid RCA (**H**). *cCTA: cardiac computed tomography angiography; LAD: left anterior descending; RCA: right coronary artery; FFR_CT_: fractional flow reserve from computed tomography; ICA: invasive coronary angiography.*

**Figure 5:**
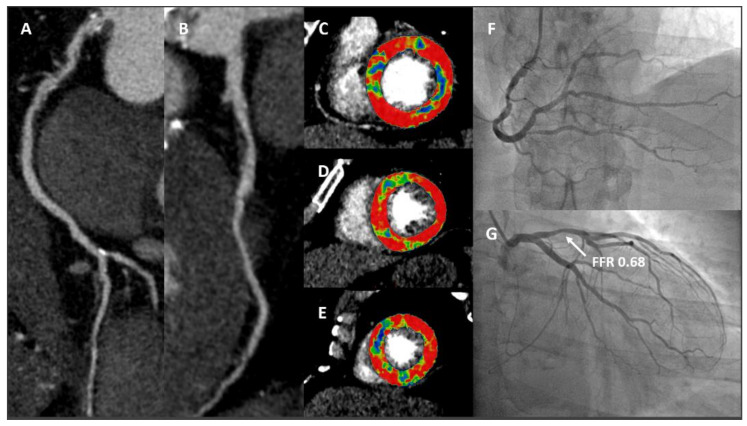
A 61-year-old male smoker with hypertension and dyslipidaemia recently developed exertional chest pain. A cCTA showed severe stenosis at the proximal RCA (Panel (**A**)) and moderate stenosis (60–70% of lumen area reduction) at the proximal LAD (Panel (**B**)). Panels (**C**–**E**): stress CTP acquired with dynamic acquisition revealed hypoperfused segments after adenosine injection at the mid to apical anterior and anteroseptal walls, basal inferolateral wall and mid to apical inferior and inferoseptal walls. Panels (**F**,**G**): ICA showing severe stenosis at the proximal RCA (**F**) and moderate stenosis at the LAD (**G**) with abnormal invasive FFR values. *cCTA: cardiac computed tomography angiography; RCA: right coronary artery; LAD: left anterior descending; CTP: computed tomography perfusion; ICA: invasive coronary angiography; FFR: fractional flow reserve.*

Either with FFR_CT_ or Stress CTP, current evidence clearly highlights that integration of anatomical and functional analysis using a single imaging method is feasible, accurate and safe. Figure 4 and Figure 5 illustrate case examples.

Compared to FFR_CT_, data from multicentre randomised trials regarding clinical management, cost-effectiveness and outcome of stress CTP are lacking. The CTP-PRO (impact of stress Cardiac computed Tomography myocardial Perfusion on downstream resources and PROgnosis in patients with suspected or known coronary artery disease) study has been designed to fill this gap [108].

## 5. Proposed Management Algorithm for Patient with Suspected CAD

As recommended by recent European guidelines, diagnostic test performance for the assessment of a patient with newly developed chest pain can be safely deferred if pre-test probability is very low (i.e. <5% according to recently proposed chart). Conversely, these guidelines suggest to choose cCTA as first test in case of low clinical suspicion and non-invasive functional tests for greater levels of pre-test probability [6]. Obviously, also site-specific expertise and test availability are considered for the diagnostic test selection.

However, the evidence summarised in the previous paragraphs clearly highlights the pivotal role gained by cCTA in the management of patients with suspected CAD and pre-test probability other than very low. Importantly, this approach is feasible when the technique is easily and rapidly available, with access to the last-generation scanners (good diagnostic performance and minimised radiation exposure), with affordable cost by the patient or local health system, and with highly trained physicians [109,110].

**Figure 6:**
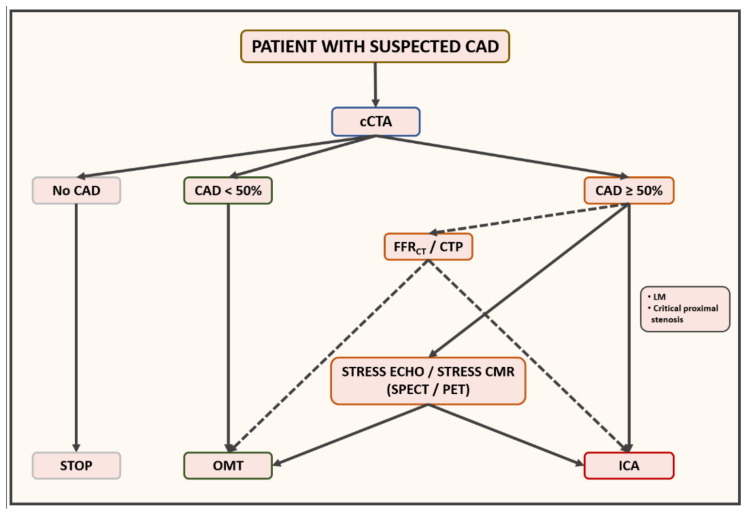
In the majority of cases, a patient with recent onset of suspected stable chest pain should be assessed with cCTA first. If no CAD is detected, other causes of chest pain should be investigated. In case of non-obstructive CAD, this result is unlikely related to symptoms, but pharmacological preventive medications (statins and antiplatelet agent) are suggested for their prognostic implications. In case of obstructive CAD, unless critical stenosis of the left main or proximal segments that require direct referral to ICA, functional evaluation should be performed as gatekeeper to invasive assessment. If available, CT-based functional assessment is suggested (FFR_CT_ or stress CTP) for a “one-stop shop approach”. Otherwise, stress echocardiography, especially in quite fit patients, or stress CMR are indicated. Finally, according to local expertise, also SPECT or PET can be prescribed in this setting. *cCTA: cardiac computed tomography angiography; CAD: coronary artery disease; ICA: invasive coronary angiography; FFR_CT_: fractional flow reserve from computed tomography; CTP: computed tomography perfusion; CMR: cardiovascular magnetic resonance; SPECT: single-photon emission computed tomography; PET: positron emission tomography.*

In an ideal scenario in which all aforementioned elements are present, we believe that the management algorithm proposed in Figure 6 can be applied to the majority of cases. A patient with recent onset of chest pain, once acute coronary syndrome and a very high likelihood of CAD (settings in which a direct referral to ICA is recommended in the former case and reasonable in the latter, respectively) is excluded, should be evaluated with cCTA first. If no atherosclerosis is detected, patient should be reassured about coronary conditions, no other tests for this specific clinical suspicion should be prescribed at least for the following 5 years [84,111], and other causes of chest pain should be investigated. If non-obstructive atherosclerosis is detected, the likelihood of relationship between coronary disease and symptoms is very low, so also in this scenario no other tests should be prescribed in the short term; however, due to the presence of coronary atherosclerosis, it’s fundamental from a prognostic point of view not only to reinforce adherence to a healthy lifestyle, but also to pursue a substantial lowering of blood cholesterol levels through statin administration, with the goal low-density lipoprotein cholesterol (LDL-C) < 70 mg/dl, usually coupled with antiplatelet therapy [112]. If obstructive stenoses are detected, a direct referral to ICA is indicated in cases of critical stenoses located at the left main and proximal segments of the three main arteries (left anterior descending, left circumflex and right coronary arteries), while a functional evaluation as gatekeeper for invasive assessment is suggested in case of moderate or severe stenoses involving non-proximal segments. The following diagnostic management in the latter setting is the most influenced by local environment. If available, CT-based FFR assessment is suggested, as up to two thirds of ICA can be cancelled without detrimental effects on patient’s prognosis [96,97,113]. In case of unavailability of CT-based FFR assessment, stress CT perfusion should be performed on top of cCTA, as with this “one-stop-shop” test both anatomical and functional information could be obtained, with high diagnostic accuracy [105]. A CT-based sequential approach could be proposed in cases of equivocal CT-based FFR results, as stress CT perfusion is characterised by high specificity and positive predictive value [107]. To minimise biological burden, CTP is suggested if a last-generation scanner (either with whole-heart coverage or dual source technologies) is available. If advanced CT tools are not available, patient should be considered for stress echocardiography (SE) or stress CMR. SE should be preferred in quite young and fit adults, as physical exercise integrated in a comprehensive protocol is able to accurately detect ischaemia and stratify prognosis [29]. Moreover, having in mind the results from ISCHEMIA Trial, SE should be preferred because with this technique regional wall motion abnormalities (RWMA), ‘heavy’ signs of ischaemic burden, are detected. If SE could not be performed due to poor acoustic windows or limitation to physical exercise, stress CMR should be performed, in light of its great diagnostic role, excellent prognostic stratification and a comprehensive myocardial evaluation. On top of these aspects, both SE and stress CMR are biologically neutral, which should be taken in account for serial tests. Finally, if above-mentioned techniques are not available, SPECT and, to a lesser extent, PET are proposed.

## 6. Conclusions

Coronary atherosclerosis is the most common cardiovascular disease and the prompt identification of extent and functional relevance of coronary plaques is crucial for the correct clinical management.

Cardiovascular imaging techniques dramatically changed knowledge regarding physiological aspects, disease progression and myocardial implications of CAD. For this reason, all imaging techniques, with their respective strengths and pitfalls, must be kept in mind when a patient with chest pain is evaluated. In this scenario, cCTA is emerging as the pivotal technique for the first step of the patient’s management, the identification of coronary plaques, and initiation of prognostically important preventative therapeutics. Then, functional techniques should be employed to identify hemodynamically significant stenosis related to patient’s symptoms and future clinical events.
